# The Relationship between Particulate Pollution Levels in Australian Cities, Meteorology, and Landscape Fire Activity Detected from MODIS Hotspots

**DOI:** 10.1371/journal.pone.0047327

**Published:** 2012-10-11

**Authors:** Owen F. Price, Grant J. Williamson, Sarah B. Henderson, Fay Johnston, David M. J. S. Bowman

**Affiliations:** 1 School of Biological Sciences, University of Wollongong, Wollongong, New South Wales, Australia; 2 School of Plant Sciences, University of Tasmania, Hobart, Tasmania, Australia; 3 Environmental Health Services, British Columbia Centre for Disease Control, Vancouver, British Columbia, Canada; 4 Menzies Research Institute, University of Tasmania, Hobart, Tasmania, Australia; Pacific Climate Impacts Consortium, Canada

## Abstract

Smoke from bushfires is an emerging issue for fire managers because of increasing evidence for its public health effects. Development of forecasting models to predict future pollution levels based on the relationship between bushfire activity and current pollution levels would be a useful management tool. As a first step, we use daily thermal anomalies detected by the MODIS Active Fire Product (referred to as “hotspots”), pollution concentrations, and meteorological data for the years 2002 to 2008, to examine the statistical relationship between fire activity in the landscapes and pollution levels around Perth and Sydney, two large Australian cities. Resultant models were statistically significant, but differed in their goodness of fit and the distance at which the strength of the relationship was strongest. For Sydney, a univariate model for hotspot activity within 100 km explained 24% of variation in pollution levels, and the best model including atmospheric variables explained 56% of variation. For Perth, the best radius was 400 km, explaining only 7% of variation, while the model including atmospheric variables explained 31% of the variation. Pollution was higher when the atmosphere was more stable and in the presence of on-shore winds, whereas there was no effect of wind blowing from the fires toward the pollution monitors. Our analysis shows there is a good prospect for developing region-specific forecasting tools combining hotspot fire activity with meteorological data.

## Introduction

An increasingly important issue of fire management revolves around the health impacts of smoke pollution. Smoke is a complex mixture of particulate and gaseous pollutants [1] that has been associated with a wide range of adverse health outcomes [2]. Smoke from bushfires can travel vast distances to affect towns and cities far from its original source [3], [4]. Bushfire smoke has been clearly associated with exacerbation of respiratory illnesses, increased respiratory hospital admissions, and visits to emergency departments [5]. The effect of bushfire smoke on other health outcomes such as cardiovascular morbidity and mortality has been less extensively researched. Of six studies into smoke-related particulate-matter mortality, three found an association [6], [7], [8], while associations with cardiovascular disease have rarely been reported [2]. However, the weight of evidence suggests that smoke particles elicit toxicological effects similar to those of particles from urban pollution (eg motor vehicle emmissions) [9], [10], [11], and the association between urban particles and respiratory and cardiovascular morbidity and mortality is well established [12].

Fire managers are required to make many decisions based on assessments of risks related to many variables (ecological, property, human safety and health), and each decision may impact other management variables. One of the greatest trade-offs is the use of prescribed burning to limit the extent and intensity of uncontrolled wildfires. For example, the 2009 Victorian Bushfire Royal Commission recommends increasing the level of burning of public bush land from 1% per year currently to 5% [13]. Most recent wildfire enquiries in Australia have made similar recommendations [14], [15]. In the USA, there is mounting pressure to increase prescribed burning rates to counter the increasing area burnt by wildland fires [16], [17], [18]. Recent evidence suggests that such increases to prescribed fire will actually increase the total area burnt, because several hectares of prescribed fire are required for each hectare of wildfire area reduction [19], [20]. Such a marked increase in prescribed burning may have adverse impacts on urban airsheds, counteracting the fact that prescribed fires have less extensive smoke plumes than wildfires [21].

Resolving this management trade-off associated with prescribed burning requires understanding of the risks associated with smoke pollution, and tools for reliably predicting where smoke is likely to travel. With appropriate warning, susceptible people can minimize their exposure by taking appropriate precautions. In addition, managers could use predictive models to decide whether or not the atmospheric conditions suit a prescribed fire, because they have a legal responsibility to avoid causing exceedances of the Australian air quality standards for particulate matter. The regulatory standard for PM_10_ (particulate matter <10 µm in aerodynamic diameter, in µg/m^3^) is currently 50 µg/m^3^ while the advisory reporting standard for PM_2.5_ (particulate matter <2.5 µm in diameter, in µg/m^3^) is 25 µg/m^3^ [22]. The standards for ambient air pollution are currently being reviewed and it is possible that the PM_2.5_ standard will be upgraded from an advisory to a regulatory standard. As PM_2.5_ is the major component of particular matter in bushfire smoke, prescribed and wild fires could become an important cause of failure to achieve the regulatory standards [23].

Most methods for tracking smoke from bushfires are based on atmospheric dispersion modelling, which is extremely data and resource intensive. At present, such models are routinely used for smoke pollution forecasting in North America [24], but not in Australia [25]. Evaluation of these models has been limited [26], [27], but the correlation between predicted and actual pollution concentrations at monitoring sites is moderate (between 0.35 and 0.71 [26], [28]), as is the spatial overlap between predicted and observed plumes (average 12% [29]). Ultimately, they are physical models rather than empirical models. We believe there is merit in investigating the independent empirical relationship between observed fire activity and measured pollution for two reasons. First, empirical models quantify the direct link between cause and effect, rather than relying on physical models and all of their necessary assumptions. Second, the insights gained may be used to improve computationally demanding physical models.

Remote sensing has revolutionised the analysis of landscape fire activity, and satellite data may also be useful for measuring smoke transport. The Moderate Resolution Imaging Spectroradiometer (MODIS) instruments aboard the Terra and Aqua satellites (operated by the US National Aeronautics and Space Administration) provide a wide range of fire-relevant information. In the active fire product (MOD14 from Terra and MYD14 from Aqua), thermal anomalies (referred to herein as “hotspots”) are recorded at a nadir resolution of 1 km for each satellite overpass (occurring globally at approximately 02∶00, 10∶00, 14∶00 and 22∶00 local time) [30], and the Fire Radiative Power (FRP) of each hotspot is measured. This attribute provides valuable information about the rate at which a fire is generating energy, and has been directly correlated with its aerosol emissions [31], [32]. Analytic use of the FRP measurement provides a unique opportunity to better examine the air quality impacts of bushfire smoke.

Here we investigate the relationship between daily pollution levels and the daily spatial pattern of hotspot activity in the landscapes surrounding two large Australian cities: Sydney and Perth. We also investigate how atmospheric conditions affect the relationship between pollution and hotspots, because smoke from fires will only move towards cities if winds are blowing from the fire to the city, and the smoke remains in the lower levels of the atmosphere. Specifically we examine: (1) whether there is a detectable link between fire activity as measured by MODIS FRP and particulate matter concentrations in cities; (2) the spatial and temporal zone of influence of bushfire activity on city pollution; (3) the potential for FRP to be combined with weather variables for smoke pollution forecasting; and (4) the use of FRP as a tool to filter the contribution of biomass smoke to records of air pollution in urban airsheds.

## Materials and Methods

### Study Areas

**Figure 1:**
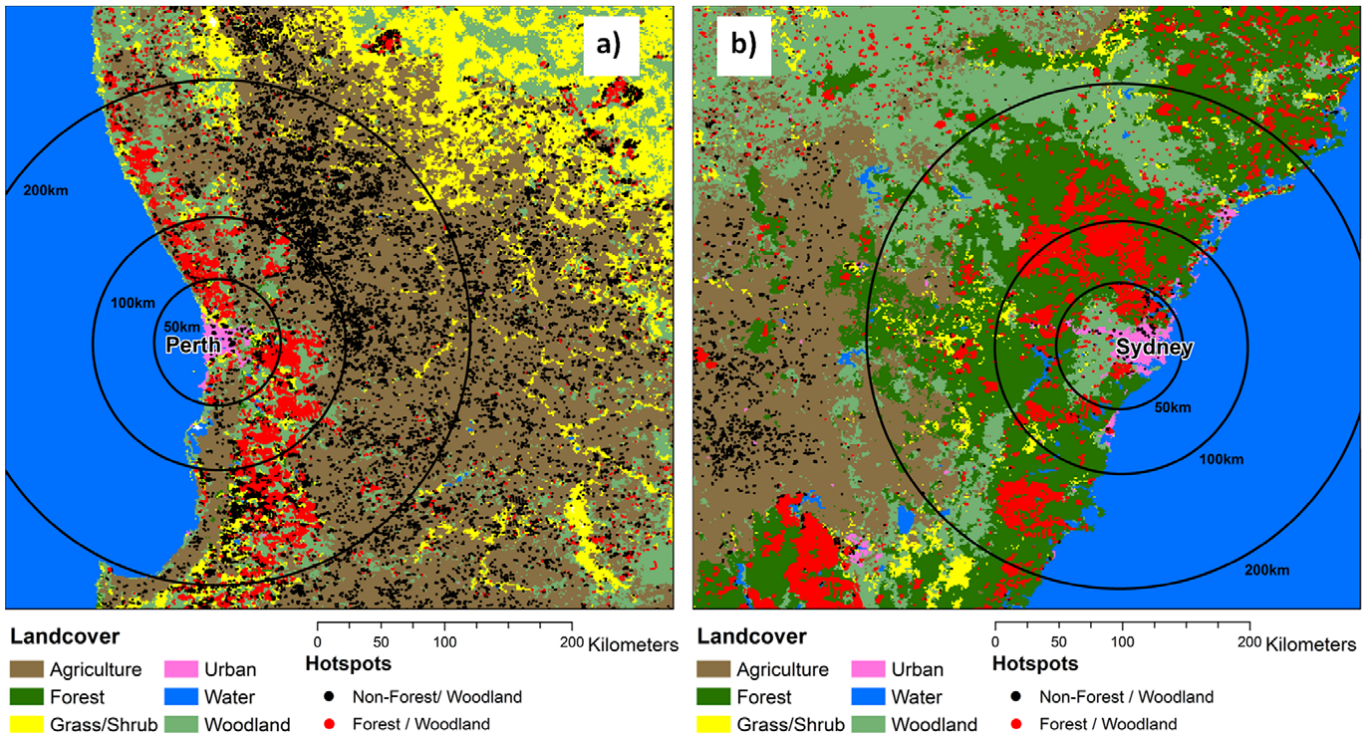
Map of the two study landscapes: a) Sydney; b) Perth. Dominant vegetation types are shaded and hotspots during the study are superimposed: forest/woodland hotspots in red; and non forest/woodland hotspots in black. Distances from the pollution monitor are shown as concentric rings.

**Figure 2:**
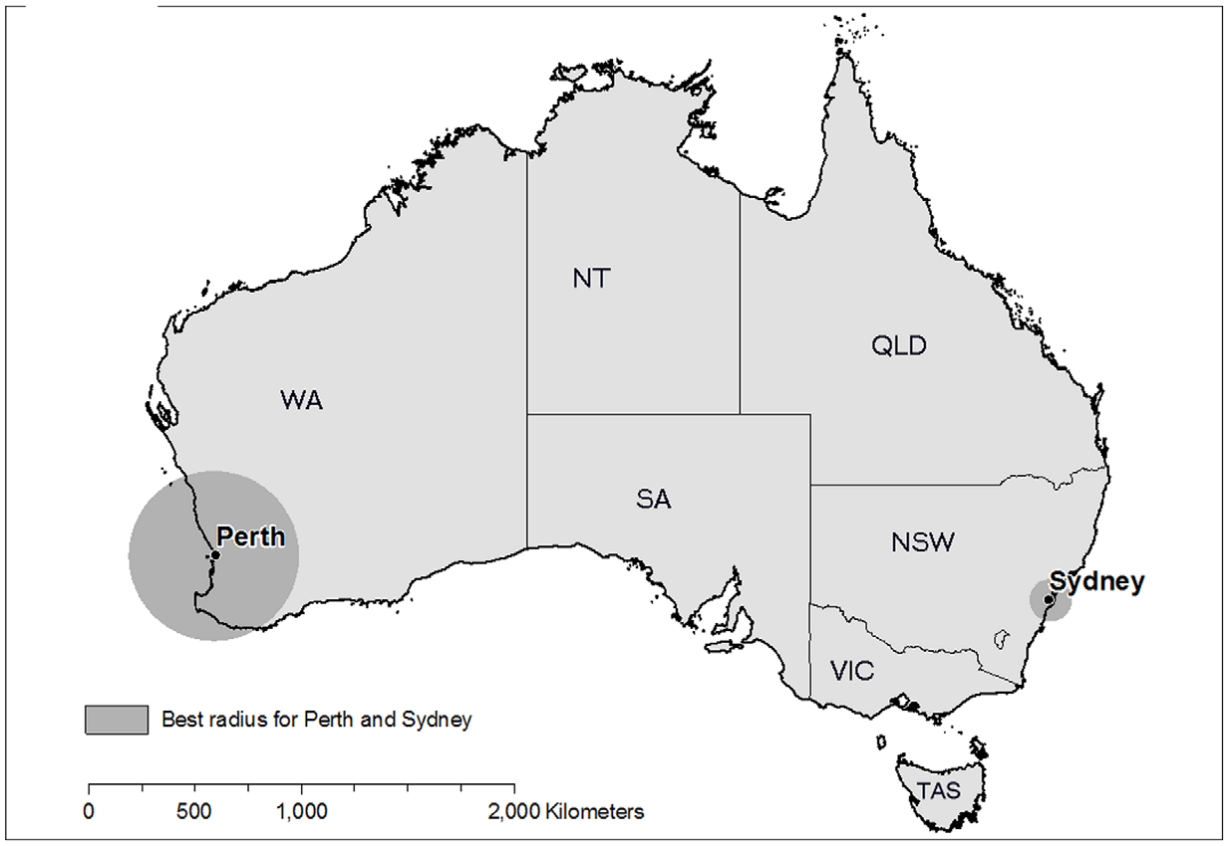
Location of Sydney and Perth, with the best radius also shown.

Sydney is a city of 4.0 million people, lying in a highly developed coastal lowland plain,surrounded by dissected sandstone tablelands (Figure 1a, Figure 2). The native vegetation in the tablelands is largely intact and is dominated by a diverse dry sclerophyll eucalypt forest [33], with a total area of approximately 20,000 km^2^. The climate is warm and temperate, and the rainfall total of 1200 mm is evenly distributed through the year (Bureau of Meteorology data). Approximately 5% of the forest is burnt by unplanned fires each year, and another 1% is burnt by prescribed fires [20]. Other flammable vegetation types (grasslands, shrublands and woodlands) are minor components and mostly further from Sydney than the forests.

Perth is the capital City of Western Australia (WA), with a population of 1.4 million people in a metropolitan area of 5300 km^2^ (Figure 2). The climate is Mediterranean, with cool wet winters and hot dry summers that are conducive to bushfires. The surrounding forest has a similar total area to Sydney (approximately 20,000 km^2^), but is much more scattered (Figure 1b). The forests are also dry sclerophyll and the communities are mostly dominated by one of two commercial species, namely jarrah (Eucalyptus marginata) and karri (Eucalyptus diversicolor) [34]. In the Warren region, typical of the forested part of WA, approximately 7% of the forest is burnt each year by prescribed fire and 1.5% by wildfire [19].

### Data

**Figure 3:**
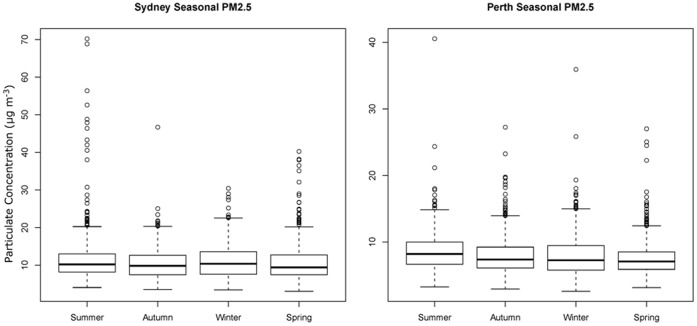
Seasonal PM_2.5_ distribution for a) Sydney; b) Perth. For each season the mean, standard deviation, 95th percentile and individual outliers are plotted. The y axis is the maximum daily PM_2.5_ (Particulate Pollution <2.5 µm in size, in µg/m^3^ ).

Pollution records were obtained from monitoring stations in each of the cities. For Sydney the monitor is at Richmond, 50 km inland (−33.618, 150.746, New South Wales (NSW) Department of Environment and Climate Change, http://www.environment.nsw.gov.au/AQMS) and for Perth it is at Duncraig, 2 km inland (−31.826, 115.783, WA Department of Environment and Conservation, WA Air Quality Data). Daily peak PM_2.5_ data was available for both cities from the beginning of 2002 to the end of 2007 (n = 2191). For Perth, the mean daily peak value across the year is approximately 8 µg/m^3^, and for Sydney it is about 10 µg/m^3^ (Figure 3). Exceedance of the 25 µg/m^3^ 24-hour air quality guideline occurs in less than 5% of days in all seasons for both cities. Exceedances are equally likely in all seasons in Perth, but for Sydney they are more frequent in summer, and these are most likely due to bushfires. The ambient level of pollution is mostly due to fossil fuel combustion [35], but other sources, including dust storms, cause some of the exceedances [36].

The daytime detects from the MODIS active fire data from Aqua and Terra were acquired from the Fire Information for Resource Management System (FIRMS – http://maps.geog.umd.edu/firms/) for 2002 through 2007. Note that Collection 4 values were used, meaning that they were not multiplied by pixel area as in Collection 5. As such, this study follows the methods developed by Ichoku and Kaufman (2005) [37] and Henderson *et al*. (2010) [38] for the use of MODIS Collection 4 data for smoke detection. For each day, all of the daytime hotspots within 500 km were identified, and their distance from and direction to the pollution monitors were calculated, and their FRP and distance-weighted FRP (FRP/distance^2^) values were recorded. The distance weighting was included because we expect smoke concentrations to diminish in proportion to the square of the distance from the source.

The daily weather record was obtained for the closest weather station from each city from the Bureau of Meteorology: Sydney Airport (BOM Station number 066037) and Perth Airport (BOM Station number 009021), which are approximately 50 km and 25 km away from the pollution monitors, respectively. We derived daily values of mean wind speed, whether the wind was onshore or not (wind angle < = 90), and Forest Fire Danger Index (FFDI). The FFDI is a function of wind speed, temperature, humidity and a drought factor, and is routinely used to predict bushfire risk in Australia [39]. In addition, upper atmospheric weather data were obtained from the same weather stations. These were used to calculate the Continuous Haines Index of atmospheric stability, using temperature and dewpoint values at 700 and 850 hPa [40], as well as the wind direction and wind speed at the surface and 700 hPa. These data are recorded several times per day, and through preliminary analysis we found that the early morning record (6 am) provided the best relationships with pollution levels. We also calculated the wind-angle relative to the dominant direction of hotspots using both the station and upper atmospheric wind directions. That is, the mean bearing between hotspots and the pollution monitor was calculated, and this was subtracted from the wind-direction so that a value of 0 represents the situation where the monitor is directly downwind from the mean centre of hotspots, and 180 where the monitor is upwind. For this calculation, all hotspots within 100 km of Sydney and 400 km of Perth were used, because these were found to be the distances over which hotspots influenced pollution levels (see below).

### Lag Effects and Duration of Pollution Events

If fire activity is to be used in a pollution forecasting tool, then it is necessary that the fire activity from one day is related to the pollution level from the following day. That means either it must take one day for the smoke to travel to the city, or otherwise the pollution event must last more than one day. It is also possible that this lag effect lasts more than one day. To explore the nature of lag effects, we constructed generalised linear models of PM_2.5_ (dependent) against FRP values (predictor) for different lag periods from 1 to 5 days. Three different lagged FRP measures were tested at each lag duration: the lag day on its own (Lag FRP), all days from the lag to the day before the current day (Combined Forecast FRP) and all days including the current day (Combined FRP). The models were structured with a normal error distribution and identity link function. The raw values of PM_2.5_ and the residuals of the model were inspected for normality, and the goodness of fit was compared among the variables using the proportion of variance explained (adjusted r^2^). To explore how long smoke may persist, we calculated the lengths of periods when the respective 95th percentile values were exceeded for each of FRP and PM_2.5_. This generated two frequency distributions of duration. If we define an “event” as any day above the 95th percentile among all days, the sum of the number of events and their durations will be approximately 109 (5% of the total number of days). For both of these analyses, the distance-weighted FRP within the best-fitting radius around the pollution monitor was used (see below, which for Sydney was 100 km and for Perth 400 km).

### Radius

There is a limit to the distance at which smoke from fires will spread to a city. Wise (2008) [4] acknowledged this in his study comparing pollution with fire activity in Texas, by using a 45 km cut-off, though he did not know the actual extent of the influence. To investigate how distance influenced pollution, we constructed generalised linear models of the relationship between FRP (predictor) and PM_2.5_ (dependent) using subsets of the hotspots at increasing radii from the monitor: at 25, 50, 75, 100, 150, 200, 300, 400 and 500 km. These were inclusive subsets, meaning that all of the hotspots closer than each radius were included. As with the lag analysis, the models used a normal error distribution, and the best interval was identified using the proportion of variance captured by the model (adjusted r^2^). For each radius, we also determined whether the r^2^ could be improved by log-transforming the FRP, PM_2.5_, or both variables. For all analyses, we used the mean FRP from the current and previous day (Combined FRP with lag = 1, which we now term FRP_01_), to take account of residual smoke from the previous day’s fires. The lag analysis (above) identified this to be the best FRP variable for predicting PM_2.5_. Also, days without fires on both days (zero FRP_01_) were excluded, giving a final sample size of 1845 days for Sydney and 1687 for Perth. This was to reduce the ‘noise’ in the data because pollution from other sources still occurred on those days, but the cause was unlikely to have been bushfire. Also, from an operational perspective, a bushfire smoke prediction model is only relevant during bushfire events, and removing zero FRP_01_ days also reduced skewness in the FRP_01_ data. Exploratory analysis revealed that removing zero FRP_01_ days did not change the form of the relationships in the models but improved their goodness of fit.

### Best Model

A model was developed to maximise the explained variation in pollution levels for each city. The variables available for this process were FRP (either single day or FRP_01_), PM_2.5_ from yesterday (PM_lag_), and all of weather variables: FFDI, Haines index, wind speed, direction and angle relative to the hotspots. PM_lag_ was also included purely to improve the predictive ability of the model. The best model was identified using a model selection process, examining all of combinations of variables and selecting the one with the lowest Akaike Information Criteria (AIC) value [41]. Supported alternative models (those with ΔAIC <2) were also identified. From the best model, all two-way interactions between FRP and the other variables were tested and retained if they reduced the AIC. The importance of each variable in the final model was assessed by calculating the proportion of explained variation attributable to each variable [42]. The modelling was repeated using the log of FRP, using the log of PM_2.5_ as the dependent variable and using all days for the sample (i.e. not restricted to days with fire activity, which is the default).

### Evaluation

A database of historical causes for extreme pollution events had previously been compiled for six cities, including Perth and Sydney [36]. This was done by matching the days where pollution exceeded the 95^th^ percentile of all values against a variety of media information and empirical data sources. Probable causes could be indentified for 67% of events and, of those, 94% were confirmed as bushfire days. The raw FRP_01_ data were validated against this database by matching days that were known bushfire days and PM_2.5_ peaks to 95th percentile peaks in the FRP_01_.

## Results

**Figure 4:**
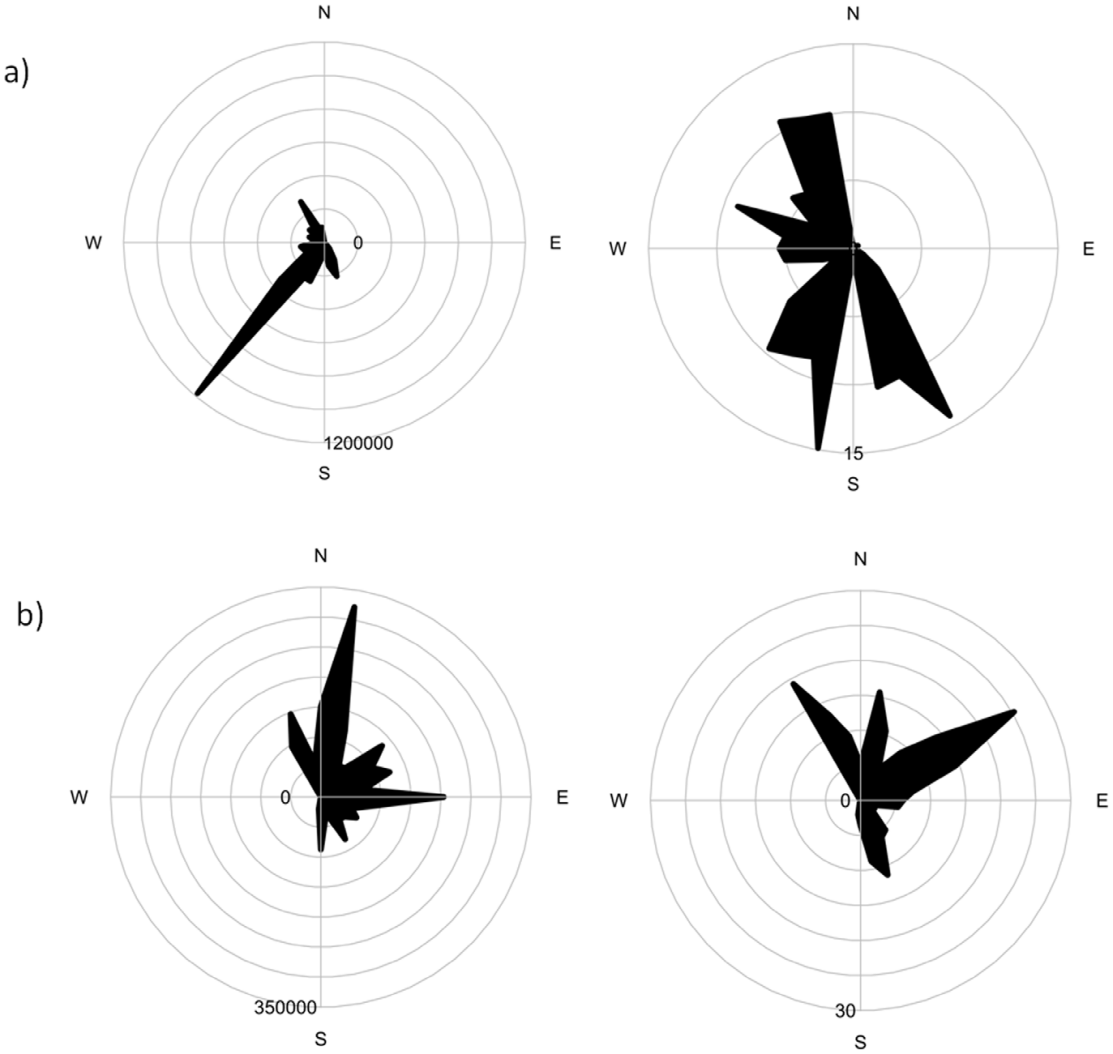
Unweighted (left) and weighted (right) hotspot rose. a) Sydney; b) Perth. The diagram shows the sum of FRP values (raw or weighted as FRP/distance^2^) in 10° sectors around the compass. The scale is in FRP units.

During the study period (2002 to 2007) 1845 and 1687 days had both pollution measures and fire hotspots in Sydney and Perth, respectively. In Sydney, the hotspots occurred in a wide arc of forest surrounding the city from north, west, and south (Figure 1a). Major, damaging fires occurred in the 2002/3 and 2006/7 fire seasons. A fire rose diagram for unweighted hotspots (showing the number of hotspots in each 10° compass sector, centred on the pollution monitor) is dominated by the fires that burnt the Australian Alps and Canberra in January 2003 (300–500 km to the south-west of Sydney) (Figure 4a). The distance-weighted fire rose shows a much more even spread of fire directions, with the fires from 2002/3 and 2006/7 prominent, but with fires from land in all directions. In Perth, hotspots were more dispersed than in Sydney and the fires were smaller (Figure 1b). Consequently, the fire roses were similar for the weighted and unweighted distances (Figure 4b). Notice the occurrence of hotspots in the direction of the ocean for Sydney, which is due to the inland position of the pollution monitor.

**Figure 5:**
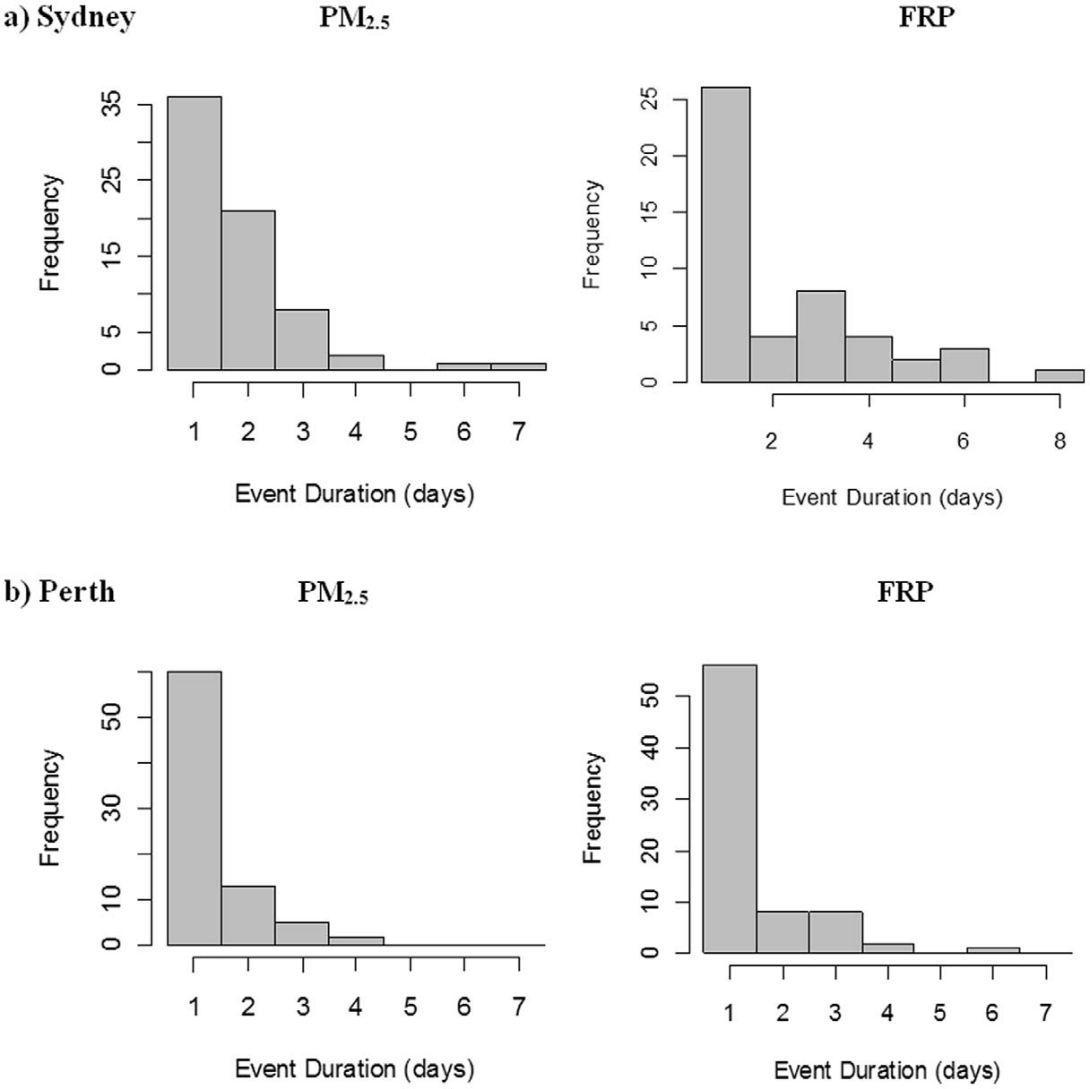
The goodness of fit between FRP and PM_2.5_ at increasing lag durations for Sydney and Perth. For each lag, three measures were compared: A) the lag day on its own (Lag FRP); B) all days including the current (Combined FRP); and C) all days from the lag to the day before the current one (Combined Forecast FRP).

In Sydney, the explanatory power of Lag FRP decreased with the length of the lag: r^2^ decreased from 0.17 for the current day to 0.04 for 4 days previously (Figure 5a). However, the best explanatory power was obtained with the Combined FRP with 1 day lag (FRP_01_, r^2^ = 0.23) and was also high for Combined FRP with 2 and 3 day lags. For the Combined Forecast FRP (excluding the current day), the explanatory power was highest for a 3 day lag (0.19). Perth showed a similar pattern, with the Combined FRP with 1 day lag giving the best explanatory power, though this was low (r^2^ = 0.84, Figure 5b). For both cities, the best FRP variable to use for modelling is the current day + previous day (FRP_01_).

Analyses on the radius of influence showed a peak in explanatory power at 100 km for Sydney (r^2^ = 0.24, Table 1). The peak for Perth was at 400 km with a lower explanatory power (r^2^ = 0.08). None of the log-transformations improved the fit of the relationships.

**Table 1:** Strength of model for various hotspot radii for Sydney and Perth.

Radius (km)	Sydney r^2^	Perth r^2^
50	0.122	0.050
75	0.161	0.046
100	0.243	0.044
150	0.183	0.045
200	0.194	0.062
300	0.181	0.072
400	0.126	0.075
500	0.112	0.062

**Figure 6:**
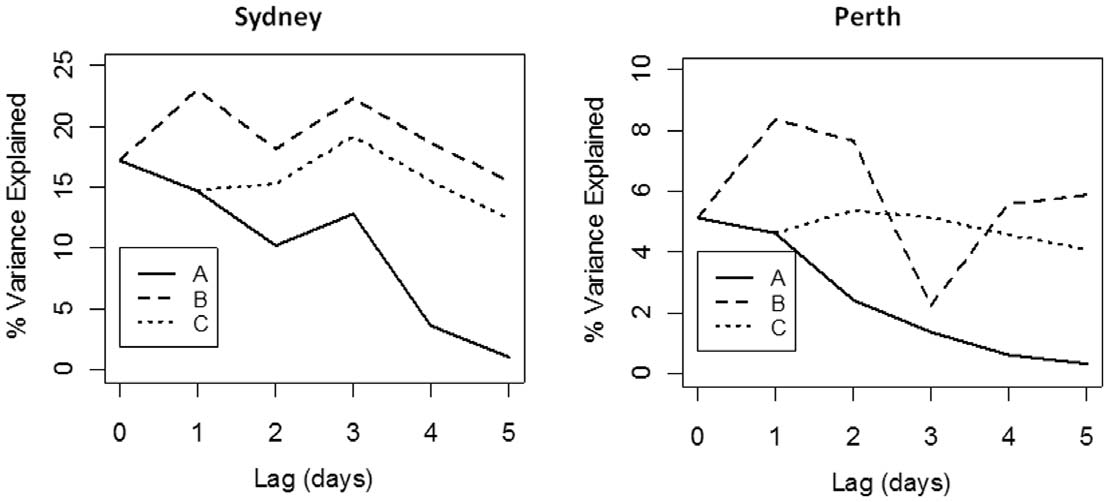
Frequency distributions of event durations for PM_2.5_ and FRP exceedances. a) Sydney and b) Perth. Values for PM_2.5_ and FRP are the 95th percentiles.

There were 69 exceedance events for PM_2.5_ in Sydney, of which 52% lasted a single day. Only four events lasted four days or more (Figure 6). The two longest events were associated with major fire events in the Sydney region (Christmas 2001 fires and early December 2002). Two other four-day events were both during winter, and were not verified fire events (11th July 2002 and 7th June 2005). In Perth, there were more events with shorter durations (n = 80, 75% lasted a single day), and only two lasted four days. These were both in May (26th May 2004 and 9th May 2006), and were not verified fire events. The exceedances in FRP showed similar patterns to the PM_2.5_ and similar differences between the cities. In Sydney there were 48 events, of which 54% lasted a single day, but ten lasted four days or more. In Perth there were 75 events, of which 75% lasted a single day, and only three were four days or more.

**Figure 7:**
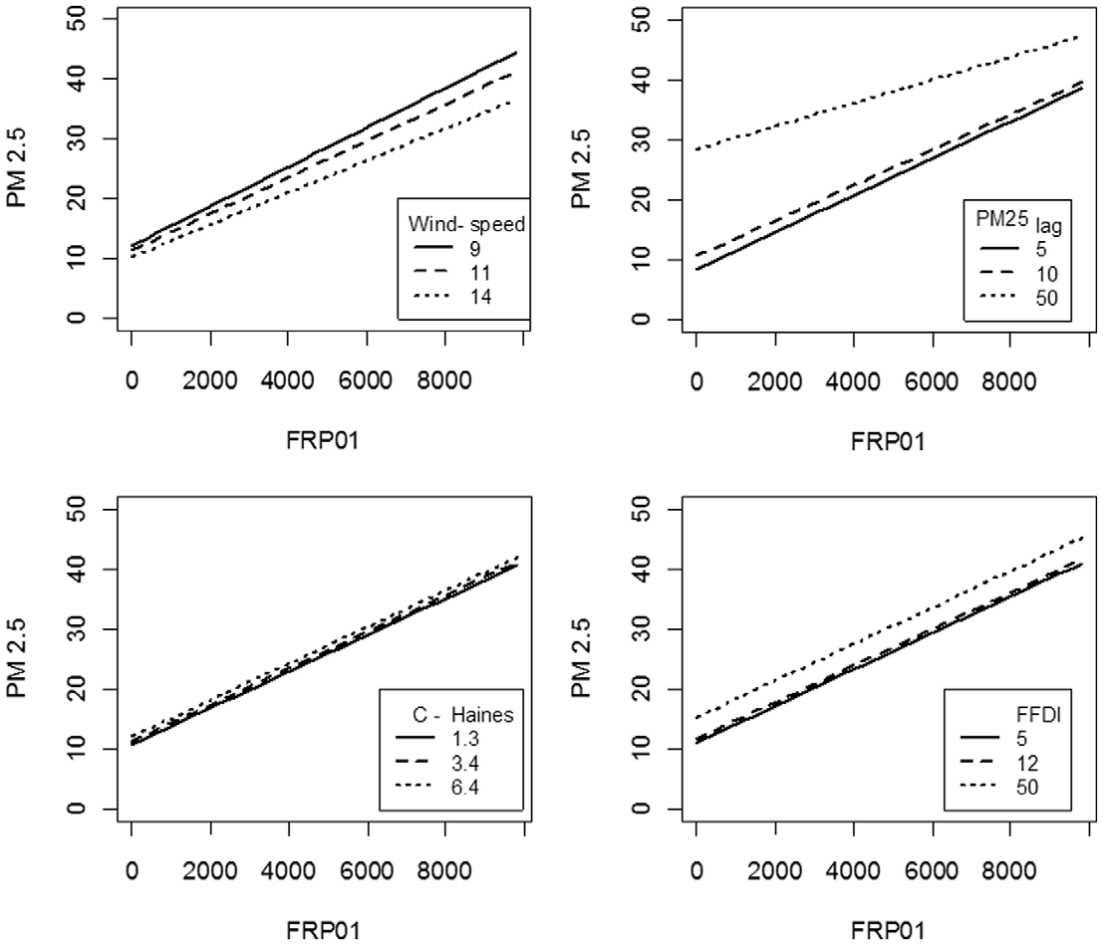
Predictions from the best model for Sydney. The plots give the relationship between PM_25_ and FRP_01_ at different levels of wind speed_sta_, PM25_lag_, C-Haines and FFDI. For wind speed_sta_ and C-Haines these levels are the quartiles (25, median, 75) while for PM25_lag_ and FFDI they are set levels (i.e. 50 represents PM_2.5_ exceedance and also a fire danger of Severe). In each plot, all other variables are held at their median values.

The best statistical model for Sydney included positive effects for FRP_01_, FFDI, C-Haines, PM_lag_ and with on-shore winds and a negative effect of wind-speed. PM25_lag_ had the strongest effect (see the importance value, Table 2a), followed by FRP_01_. There were also negative interactions between FRP_01_ and wind-speed,and FRP_01_ and PM25_lag_, but these were weak effects (Table 2, Figure 7). The model explained 56% of variance in PM_2.5_ (r^2^ = 0.562) and there were no supported alternatives. When the analysis was repeated without PM25_lag_, the final model was similar, but explained 45% of variation. Using log(PM_2.5_) as the dependent variable or including all days also resulted in similar models and explanatory power (r^2^ = 0.561, 0.569 respectively). Using the log of FRP as a predictor variable gave weaker results than the untransformed FRP variables.

**Table 2:** Final model tables.

Sydney, r^2^ = 0.562, n = 1845.					
	Estimate	Std. Err	t	P	Importance
(Intercept)	8.165	0.371	21.988	<0.001	
FFDI	0.094	0.015	6.210	<0.001	0.053
FRP_01_	0.0047	0.0004	12.473	<0.001	0.236
Wind-speed_sta_	−0.369	0.021	−17.316	<0.001	0.129
C-Haines	0.262	0.026	9.932	<0.001	0.103
On-shore_700_	0.592	0.234	2.532	0.011	0.015
PM25_lag_	0.444	0.019	23.266	<0.001	0.428
FRP_01_:Wind-speed_sta_	−1.22e-4	2.50e-5	−4.874	<0.001	0.020
FRP_01_:PM25_lag_	−2.57e-5	8.78e-6	−2.926	0.003	0.016
**Perth, r^2^ = 0.310, n = 1667.**					
	**Estimate**	**Std. Err**	**t**	**P**	**Importance**
(Intercept)	6.083	0.312	19.510	<2e-16	
FFDI	0.029	0.009	3.071	0.002	0.033
FRP_01_	−2.80e-5	8.27e-5	−0.338	0.735	0.107
Wind-speed_sta_	−0.146	0.014	−10.339	<2e-16	0.148
C-Haines	0.129	0.018	7.102	0.000	0.205
On-shore_700_	0.441	0.159	2.779	0.006	0.012
PM25_lag_	0.364	0.021	17.333	<2e-16	0.483
FRP_01_:C-Haines	3.33e-5	1.00e-5	3.316	0.001	0.013

Importance refers to Relative Importance: the proportion of explained variance attributable to each variable (sum to 1.0).

The best model for Perth was similar to Sydney, but explained less variation (r^2^ = 0.310, Table 2b). There were positive effects of FFDI, C-Haines and PM25_lag_ and on-shore wind and a negative effect of wind speed. The FRP_01_ effect was weak, and there was an interaction between FRP_01_ and C-Haines, which meant that FRP had a positive relationship with PM_2.5_ which became slightly negative when the C-Haines index was less than 1.3 (on about 31% of days). There were no supported alternative models. PM25_lag_ was the most important variable, followed by C-Haines. Without PM25_lag_, the best model explained only 19% of variance. Using log(PM_2.5_) as the dependent variable, including all days, or using log of FRP as the predictor variable all resulted in similar models with slightly lower explanatory power (r^2^ = 0.294, 0.308, 0.306 respectively).

There were 54 validated bushfire events in Sydney with matching PM_2.5_ peaks, and 74% of these corresponded with peaks in the raw FRP_01_ value and 61% corresponded with peaks in the model predictions. For Perth there were 23 events and 35% corresponded with the raw FRP_01_ and 43% with the model predictions.

## Discussion

We have demonstrated that there is a relationship between fire activity detectable from satellites and pollution in urban centres. Moreover, there are windows of spatial and temporal influence of fire activity on pollution. For Sydney, the radius of influence is relatively small (100 km) and the relationship is strong, while for Perth the radius is much larger (400 km) and the relationship is weak. The explanatory power of the models was improved by including weather and pollution history variables, to the extent that more than half of the variance in pollution could be explained for Sydney. The weather relationships revealed higher pollution when the atmosphere was stable, when wind speed was low and when the wind in the upper atmosphere was blowing on-shore. Importantly, wind flow from the fires to the monitor was not present in the models. This is presumably because on-shore winds that trap in-situ pollution in the city basins are more important to the overall level of pollution than winds transporting smoke from bushfires, even if days without fire activity are excluded. This trapping effect is probably why stable air and low wind speed also lead to more pollution.

The differences between the cities have a logical explanation. Sydney is bounded on three sides by extensive forests (approximately 20,000 km^2^) at a distance of about 100 km. Beyond this is an extensive agricultural belt with very little fire activity. The mean area burnt in these forests is approximately 5% per year, of which more than 80% is unplanned fire [20]. Generally, the wind associated with bushfire events blows from an arc between northwest and southwest, and so blows smoke into the city. The forests around Perth are much more scattered, comprising small patches spread across a larger region. A higher proportion of the forests are burnt each year (8.5%) [19], but the regime is dominated by prescribed fires (7% compared to 1.5% for unplanned fire). The closeness of the forests and the more extensive unplanned fire explain why the model has a smaller radius and better goodness of fit for Sydney than for Perth. These differences between cities mean that a forecast tool must be based on a model specific to each city.

Our empirical modelling method shows some potential for developing a forecasting tool for bushfire planning. For both cities, the best single predictor of daily pollution is the previous day’s pollution, which may be of use to health authorities. Nevertheless, the FRP and weather variables also contribute substantially to the predictive power. For Sydney, the predictive model explained most of the variation in pollution and three quarters of the known bushfire caused pollution days were identified as peaks in the FRP data. For Perth, the previous days FRP is actually a better predictor than the current day, albeit with low predictive power. Our method may be more efficient and accurate for identifying historical fires than other methods available that require laborious searches of print media and government reports [43].

Improvements could be made to the forecasting ability by addressing some of the limitations of the data. The most important of these is the lack of identification of non-bushfire sources of pollution, since these are the principle driver of pollution levels on most days. Pollution from these sources also varies and our analysis suggests that they respond to weather conditions, perhaps more than smoke does. If non-smoke pollution days could be filtered from the data then the accuracy of the models would be greatly increased. A more explicit representation of atmospheric transport such as a two or three-dimensional wind-field between the source and monitor would also improve the relationship. Many fires, especially larger ones, inject smoke high into the atmosphere which may reduce pollution on the ground [44]. Since there is actually a relationship between FRP and injection height [44], this effect could be directly incorporated into a predictive model and may be more successful than the use of an atmospheric stability index such as C-Haines. Ultimately, a hybrid model using daily snapshots of FRP and a three-dimensional atmospheric transport model may be the most fruitful approach.

There are other limitations that affect the accuracy of the models. The raw FRP values used here do not account for differences in vegetation type. Vegetation correction factors have been applied elsewhere to FRP values [37], but none have been developed for Australia. The episodic nature of major bushfire events means that the six year time series used here only captured a small number of events (54 events reported in the media for Sydney and 23 for Perth), and these varied greatly in magnitude. The analysis would be improved with a longer time series. Sydney experiences extreme fire seasons (where >10% of the forest area burns) between once and twice per decade [20], so it may be that several decades of data are required to determine the nature of the relationship between FRP and pollution levels.

Our results are also relevant to the debate over the appropriate level of prescribed fire treatment. Prescribed fire is imposed as a means of reducing the area and impact of wildfire, and has been suggested as one means of preventing increased wildfire activity due to climate change. However, in many Australian landscapes several hectares of prescribed fire are required to reduce the area of wildfire by one hectare. For example, around Sydney 3–4 ha of prescribed fire are required per hectare reduction in wildfire area [20], [45], and the forests around Perth, 6 ha are required [19]. However, prescribed fires consume less biomass per hectare than wildfires because they are more patchy [46], are usually confined to the surface fuels and leave larger proportion of fuel unburned. It has been shown for Southern Australia that smoke plumes from prescribed fires blow over cities, though on average these are five times smaller than plumes from wildfires [21]. Further examination of the contribution of prescribed and wildfires to smoke is needed before the likely impact of increased prescribed burning could be accurately predicted.

In conclusion, this study has identified a clear relationship between remotely sensed fire activity and pollution levels in two Australian cities. It has also identified the distance over which fires tend to supply smoke to the cities, and some of the challenges to developing a useful operational tool. With some improvements, models developed using this method could become useful in many fire-prone regions around the world for forecasting smoke pollution. They may be used as an adjunct to smoke-plume modelling or may eventually prove to be more useful. Even as they are now, the method can be used for rapid retrospective identification of bushfire pollution days, in contrast to the labour intensive methods used by Johnston et al. [36]. With further separation of FRP into wild and prescribed fires, our approach could also provide a new tool for understanding the comparative smoke pollution exposure to humans from fires of both types.
